# Supplementary material for: The effects of hip- and ankle-focused exercise intervention on dynamic knee valgus: a systematic review
Source: PeerJ. 2021 Jul 5;9:e11731. doi: 10.7717/peerj.11731 (PMC8265381; doi:10.7717/peerj.11731)
Supplement: Supplemental Information 2 [file peerj-09-11731-s002.pdf]

The rationale and the contribution of the current review :

A systematic review by Lima et al. (2018) evaluated the association between ankle dorsiflexion (bottom-up kinetic chain) and DKV in interventional and non-interventional studies. By including the non-interventional studies, the review results might not be related to the impact of exercises on reducing DKV. Meanwhile, a systematic review by Dix et al. (2018) investigated the association between hip muscle strength (top-down kinetic chain) and DKV in asymptomatic (i.e., free from any injury) females. Similarly, the review did not investigate the effects of exercises that contribute to hip muscle strength in reducing DKV. A narrative review by Ford et al. (2015) provided details on a hip-focused neuromuscular exercise intervention to improve DKV. However, narrative review by Ford et al., (2015) excluded in-depth and systematic literature search approach, hence it may miss out several relevant papers. To the best of our knowledge, no systematic review has been conducted that focused on the effects of top-down (hip-focused) or bottom-up (ankle-focused) kinetic chain of exercise intervention on DKV mechanisms. The review will shed light on how the exercise training programs may improve the mechanisms behind knee injury. Therefore, the present systematic review aims to determine the influence of hip- and ankle-focused exercise intervention on improving DKV.

Previous systematic reviews revealed that increased ankle dorsiflexion ROM (Lima et al., 2018) and hip strength (Dix et al., 2018) were crucial in reducing DKV. An evidence-based review found that the neuromuscular exercises targeting hip musculature activation and strength may alter the DKV (Ford et al., 2015).

In this review, we investigated the effects of exercise interventions, based on either top-down or bottom-up kinetic chains, on minimizing DKV during specific tasks. As DKV is one of the main factors that could increase the risk of lower limb injury, findings from this review highlighted the importance of prescribing exercise training program to improve DKV.

Four studies (Barendrecht et al., 2011; Bell et al., 2013; Baldon et al., 2014; Thompson-Kolesar et al., 2017) found significant changes in knee valgus following a training program that combined both top-down and bottom-up kinetic chain exercises. Three studies (Sheerin, Hume, and Whattman, 2012; Saad et al., 2018; Jeong et al., 2021) discovered that top-down kinetic chain exercise intervention programs were also effective in reducing knee valgus. However, McCurdy et al.,

(2012), Czasche et al., (2017) and Araújo et al., (2017) did not detect any significant effects of the exercise intervention in minimizing DKV. Therefore, we concluded that a combination hip- and ankle- focused exercises and hip-focused (top-down kinetic chain) exercises only are more favorable than exercise program consisted of ankle-focused (bottom-up kinetic chain) exercises only. Apart from focusing on the load and volume of prescribed exercises, the specificity of exercises should also be emphasized. These findings might help the athletes and coaches to design appropriate exercise programs in reducing DKV.
